# The Responses of Bioactive Betanin Pigment and Its Derivatives from a Red Beetroot (*Beta vulgaris* L.) Betalain-Rich Extract to Hypochlorous Acid

**DOI:** 10.3390/ijms22031155

**Published:** 2021-01-25

**Authors:** Karolina Starzak, Katarzyna Sutor, Tomasz Świergosz, Boris Nemzer, Zbigniew Pietrzkowski, Łukasz Popenda, Shi-Rong Liu, Shu-Pao Wu, Sławomir Wybraniec

**Affiliations:** 1 Department of Analytical Chemistry, Faculty of Chemical Engineering and Technology, Cracow University of Technology, Warszawska 24, 31-155 Kraków, Poland; karolina.starzak@pk.edu.pl (K.S.); katarzyna.sutor@doktorant.pk.edu.pl (K.S.); tomasz.swiergosz@pk.edu.pl (T.Ś.); 2 Research and Analytical Center, VDF FutureCeuticals, Inc., 2692 N. State Rt. 1-17, Momence, IL 60954, USA; bnemzer@futureceuticals.com; 3 Food Science & Human Nutrition, University of Illinois at Urbana-Champaign, 260 Bevier Hall, 905 S Goodwin Ave, Urbana, IL 61801, USA; 4 Discovery Research Lab, VDF FutureCeuticals, Inc., 23 Peters Canyon Rd, Irvine, CA 92606, USA; zb@futureceuticals.com; 5 NanoBioMedical Centre, Adam Mickiewicz University in Poznań, Wszechnicy Piastowskiej 3, 61-614 Poznań, Poland; lukasz.popenda@amu.edu.pl; 6 Department of Applied Chemistry, National Chiao Tung University, Hsinchu 300, Taiwan; neptue7218@gmail.com (S.-R.L.); spwu@mail.nctu.edu.tw (S.-P.W.)

**Keywords:** betacyanins, betalain rich extract, inflammation, hypochlorite scavenging

## Abstract

Neutrophils produce hypochlorous acid (HOCl) as well as other reactive oxygen species as part of a natural innate immune response in the human body; however, excessive levels of HOCl can ultimately be detrimental to health. Recent reports suggest that betacyanin plant pigments can act as potent scavengers of inflammatory factors and are notably effective against HOCl. Comparison of the in vitro anti-hypochlorite activities of a novel betalain-rich red beetroot (*Beta vulgaris* L.) extract with its pure betalainic pigments revealed that the extract had the highest anti-hypochlorite activity, far exceeding the activity of all of the betalainic derivatives and selected reference antioxidants. This suggests that it may be an important food-based candidate for management of inflammatory conditions induced by excessive HOCl production. Among all pigments studied, betanidin exhibited the highest activity across the pH range.

## 1. Introduction

**Figure 1:**
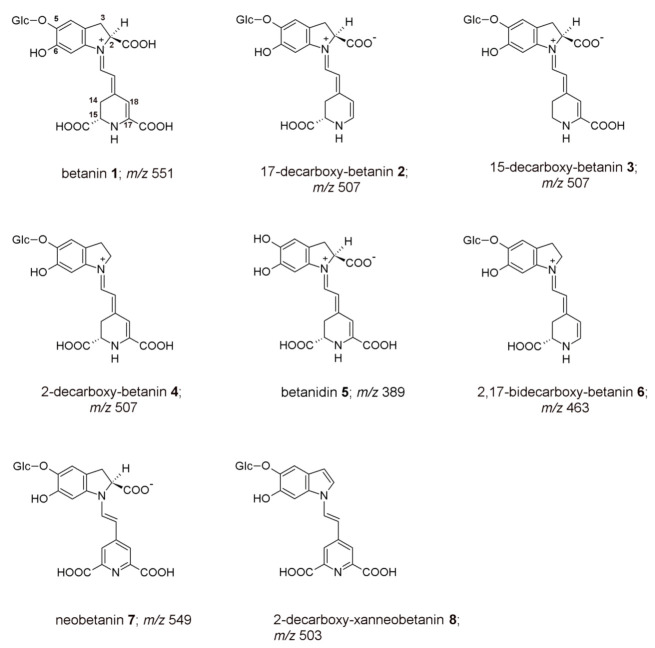
Chemical structures of betanin **1**, its derivatives (**2**–**4**, **6**–**8**), and betanidin **5** present in the BRE subjected to studies on chlorination and anti-hypochlorite activity in this contribution. Pigment **6** was tested together with its epimer **6′** (Table 1).

Betalains are natural, water-soluble pigments originating in plants across a family of the Caryophyllales order [1,2,3,4]. They are found in high concentrations in red beet (*Beta vulgaris* L.), as well as in fungi of the Basidiomycetes family, especially in red toadstool (*Amanita muscaria* L.) and in tropical cacti fruit (*Opuntia ficus-indica* L.) [1,2]. These pigments are divided into red-violet betacyanins (primarily the immonium conjugates of betalamic acid with *cyclo*-DOPA (forming betanidin) or glycosylated *cyclo*-DOPA (in other betacyanins)) and yellow-orange betaxanthins (being the immonium derivatives of betalamic acid with different amines and amino acids) [1,2,3]. A large variety of compounds belonging to the class of betacyanins results from the existence of two hydroxyl groups at carbon C-5, or C-6 (catechol moiety) in the betanidin 5 structure (Figure 1). These hydroxyl groups are readily *O*-glycosylated leading to the formation of 5-*O*-glycosides such as betanin 1 or 6-*O*-glycosides such as gomphrenin I [1,2,3]. 

The attributes of betalains as natural colorants provide a wide spectrum of applications as natural additives in the food industry [1,2,3]. It is worth noting that no adverse effects have ever been reported due to the ingestion of betalains by living organisms [4]. Betalain pigments have the ability to bioaccumulate in blood and yet are generally excreted from the body via the urine within a few hours after ingestion [3,4]. Besides being extensively used in the food industry as natural food colorants, betalains exhibit strong antioxidant and chemopreventive activities [1,2,3,4]. These characteristics contribute to the increased interest in betacyanins, especially from *B. vulgaris* root, in oncology applications [3,4]. Furthermore, recent studies indicate that betalains can act as potent scavengers of inflammatory factors [5,6] and may improve various health conditions related to inflammation [7,8]. Hypochlorous acid (HOCl) is an important reactive oxygen species (ROS) that causes oxidation and chlorination reactions. It is produced by activated neutrophils and monocytes via the reaction of H_2_O_2_ with Cl^−^ ions catalyzed by the heme enzyme myeloperoxidase [9,10]. In living systems, HOCl can react at the molecular level with primary amines and other *N*-compounds, leading to the formation of chloramines and N-Cl derivatives [11]. The presence of HOCl and its derivatives in intercellular spaces can lead to the local irritation of epithelial tissue cells, damage of proteins, nucleotides, DNA, RNA, fatty acids, or cholesterol [11,12,13,14,15]. 

Although neutrophilic production of HOCl is part of a natural innate immune response, excessive HOCl levels can ultimately be detrimental to health. The harmful impact of hypochlorite derivatives occurs in people suffering from chronic inflammation, e.g., joints. Overproduction of HOCl during inflammation may also cause or enhance the pathogenesis of various diseases including Alzheimer’s disease, cardiovascular disease, atherosclerosis, organ transplant rejection, and even certain cancers [11]. In individuals with joint distress, locally elevated concentrations of hypochlorous acid and other ROS contribute to the stimulation of the immune system and to the increased production of antibacterial compounds [7,8,12]. Allegra et al. reported the effectiveness of betanin and indicaxanthin in scavenging hypochlorous acid [16]. 

Previously, a novel betalain-rich extract/concentrate (BRE) was tested in a pilot clinical study that reported short-term treatment with BRE improved the function and comfort of knee joints in individuals with knee distress [7,8]. Said BRE was designed to deliver the benefits of betalains without the accompanying sugars, nitrates, and calories from beetroot, and is currently being used as a dietary supplement [17]. During extraction, naturally occurring sugar derivatives and nitrates are selectively eliminated in order to yield a standardized minimum weight content of 25% betalain. BRE contains mainly betanin, neobetanin, and some decarboxylated derivatives [17]. 

Another study indicated that betalains are readily chlorinated in the presence of HOCl at pH 3–5; however, when the pH of the reaction mixture is increased, the oxidation of betalains prevails [5,6]. Therefore, the ability of betalains to react with hypochlorous acid within a wide pH range [5,6] suggests the possible use of BRE as a dietary supplement for the support of healthy scavenging of HOCl in vivo. Recently, we worked to prove the hypothesis of the betacyanin chlorination mechanism by HOCl [5,6,18]. This was accomplished by LCMS-IT-TOF and NMR analyses of the resultant chlorination products [18]. 

In this contribution, we used fluorescent methods that employed two novel HOCl-specific BODIPY-based HCSe and HCS probes [19,20] to study the in vitro anti-hypochlorite activities of selected purified betacyanins and their derivatives as well as the BRE, considering the reactivity of particular betalainic pigments depending on the structures of the active compounds.

HCSe and HCS are novel, fluorescent probes based on the BODIPY skeleton. They are sensitive to hypochlorous acid. These probes produce no fluorescence in acetonitrile/aqueous solutions in the absence of hypochlorous acid. The addition of HOCl to a reaction mixture induces a selective oxidation of the probes, leading to the formation of strong fluorescent derivatives HCSeO or HCSO, respectively [19,20]. An important characteristic of the probes is that they are highly sensitive to hypochlorous acid at pH 7.4 and are able to penetrate the membranes of living cells. This allows for their utilization in in vitro assays that simulate inflammatory processes due to the presence of HOCl and OCl^-^ at a physiological pH of 7.4 [19,20]. 

## 2. Results and Discussion

**Figure 2:**
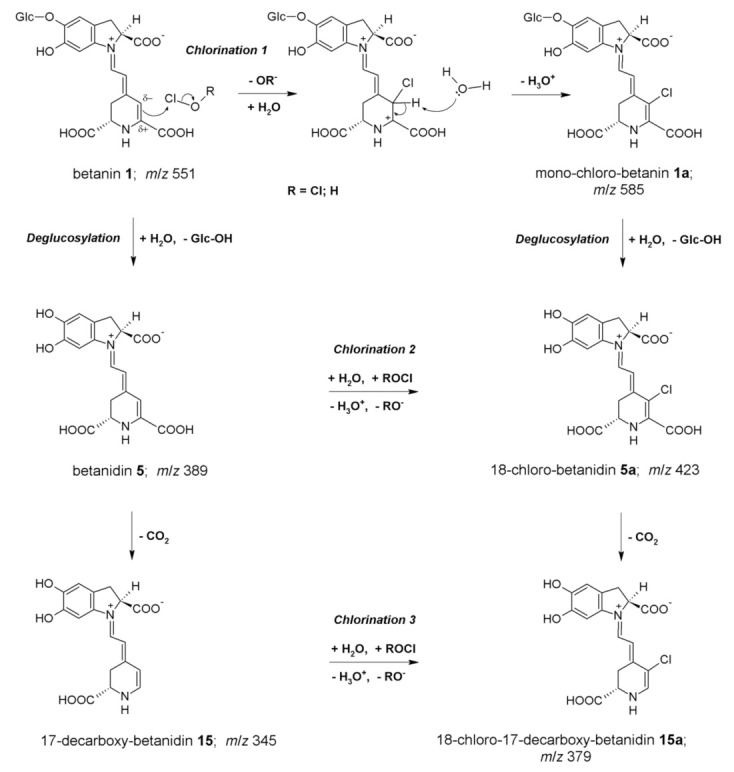
Proposed mechanism of betanin chlorination induced by NaOCl [5] as well as further pathways of deglucosylation and decarboxylation.

Our previous studies on the mechanism of betacyanin chlorination indicated that the formation of monochlorinated betanin and betanidin (as well as their respective decarboxylated derivatives) resulted from the reaction of betacyanins with HOCl or Cl_2_O. Cl_2_O is a potent chlorinating agent and coexists with HOCl in equilibrium, especially in acidic conditions [5,6,18]. The experiments were monitored by HPLC with low-resolution mass spectrometric detection. These chemical changes were studied also by the enzymatic chlorination of betacyanins catalyzed by MPO at NaCl concentration (150 mM—typical to normal physiological levels), enabling a slow and continuous production of HOCl from Cl^−^ [5]. The chlorination mechanism and the position of the electrophilic substitution in betacyanins (Figure 2), as well as their molecular formulas, were studied using detailed mass spectrometric experiments on the fragmentation of the chlorinated pigments performed by the high-resolution IT-TOF technique [5,6,18]. The reactions with HOCl and Cl_2_O were proposed (Figure 2) according to an electrophilic mechanism based upon the departing group ability from Cl^+^ in HOCl (-OH^−^) and in Cl_2_O (-OCl^−^) [5,18]. This is also supported by the inactivity of neobetanin towards chlorination. Neobetanin is an aromatized betanin with a pyridine ring resulting from betanin oxidation. The fact that this ring cannot be chlorinated suggests that in betacyanins, only the unsaturated bond is attacked, preferably at C-18 due to its partial negative charge [5]. The mass spectrometric studies were further supported by additional structural analyses using NMR techniques that completed the identification of the chlorinated betacyanins [18]. 

In this study, a comparison of chlorination kinetics was conducted during measurements of the in vitro anti-hypochlorite activity of pure isolated betalainic pigments from BRE. Earlier comprehensive characterization of the BRE [17,21] enabled isolation of betalainic pigments and further study on the chlorination/oxidation of betanin as well as its derivatives for elucidation of different reaction mechanisms [5,6,18]. As mentioned previously [17], the Nilsson calculation method [22] of betalain total concentration is related to betacyanins and betaxanthins only when there is a lack of their derivatives in the formulations. Therefore, the calculated results for the yellow components of the BRE roughly represent the following group of betalains: betaxanthins, neobetanin as well as decarboxylated betacyanins, neobetacyanins, and xanneobetacyanins [17]. This is important when establishing correlations between concentrations of betalains in complex mixtures and their respective activities, such as anti-hypochlorite activity or free-radical scavenging capacity as measured in this study.

This contribution reports on their anti-hypochlorite activity also compared to their antioxidant activity [23]. 

### 2.1. Characterization of Reactive Betalainic Pigments of B. vulgaris Concentrated Extract 

A previous study resulted in a tentative characterization of the most concentrated decarboxylated and dehydrogenated derivatives of betacyanins present in the BRE [17]. Said compounds’ identities were assumed based upon: (a) presumptions derived from the proposed oxidation pathways [24,25,26] of betanin and its derivatives; (b) on a comparison of their absorption and chromatographic properties [24,25,26]. Following the determination of actual betalain structures, anti-hypochlorite activity measurements were performed for isolated betanin **1**, 17-decarboxy-betanin **2**, 15-decarboxy-betanin **3**, 2-decarboxy-betanin **4**, betanidin **5**, 2,17-decarboxy-betanin/-isobetanin **6/6′**, neobetanin **7**, and 2-decarboxy-xanneobetanin **8** (Figure 1, Table 1) [17]. For comparison, the same measurements were performed on selected, commonly known antioxidants including: ascorbic acid **9**, caffeic acid **10**, catechin **11**, and quercetin **12** as well as for the BRE **13** and Trolox **14**. 

In order to simplify the nomenclature, we propose the substitution of the phrase “xan” instead of “2,3-dehydro” in the trivial name of the 2,3-dehydrogenated betacyanins in accordance with our previous contribution [26]. Such a simplification is similar to the “neo” prefix used for several decades to replace the cumbersome “14,15-dehydro”. The “xan” prefix underlines the hypsochromic effect observed after dehydrogenation of betacyanins that leads to the formation of yellow pigments. Therefore, because betacyanins can appear as both 2,3- and 14,15-dehydrogenated, there is a group of pigments formed that can be called “xanneobetacyanins”. In the BRE, three compounds from such a group were tentatively detected, together with four neobetacyanins [17]. 

The single chromatographic peak of 15-decarboxy-betanin **3** (Figure 1) with an absorption maximum at λ_max_ 528 nm is very well separated from 17-decarboxy-betanin/isobetanin **2/2′** and 2-decarboxy-betanin/-isobetanin **4/4′** also present in the extract [17]. The enrichment of 15-decarboxy-betanin **3** in the BRE is at a similar level as for **2/2′**, suggesting the same formation rate; therefore, it is one of the main decarboxylation derivatives of betanin formed during the preparation of the extract. The most hydrophobic product of betanin degradation/oxidation is 2-decarboxy-xanneobetanin **8**, characterized by a single chromatographic peak, indicating the lack of chirality at carbon C-15, and by the absorption maximum at λ_max_ 422 nm. From the point of view of oxidation studies, this is an important compound because it is generated from betanin, most likely via an unstable quinone methide intermediate and the fairly stable 2-decarboxy-2,3-dehydro-betanin (2-decarboxy-xanbetanin) [24,25], which is oxidized in the second step to a much more stable 2-decarboxy-xanneobetanin **8** (Figure 1). 

### 2.2. Chlorination of Betanin by Sodium Hypochlorite 

**Figure 3:**
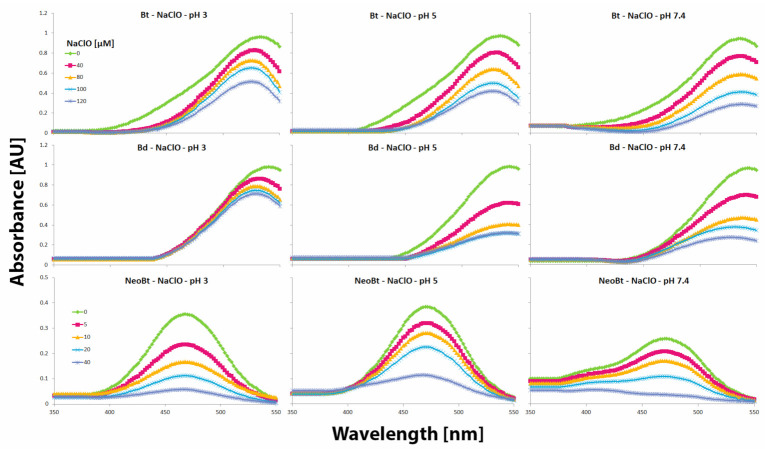
The spectra obtained after 10 min spectrophotometric tracing of betanin **1** (Bt), betanidin **5** (Bd), and neobetanin **7** (NeoBt) reaction progress induced by NaClO.

The results obtained after 10 min spectrophotometric tracing of betanin **1**, betanidin **5**, and neobetanin **7** reaction progress induced by NaClO (Table 2, Figure 3) reveal the differences between the partially oxidized betacyanin (neobetanin **7**) which does not undergo chlorination [5] and non-oxidized betacyanins (**1** and **5**) which are easily chlorinated at pH 3. The chlorination effects are detected by the hypsochromic shift of absorption maximum λ_max_ from ca. 538–540 nm (for **1** and **5**) to 522–524 nm typical of chlorinated betacyanins. This effect is not observed for neobetanin **7** and is also diminished at higher pH (5 and 7.4) for **1** and **5** (Figure 3). In those cases, the main reaction processes involve oxidation of the pigments, presumably by HClO or other chlorine species being in equilibrium in the reacting solution [5].

**Figure 4:**
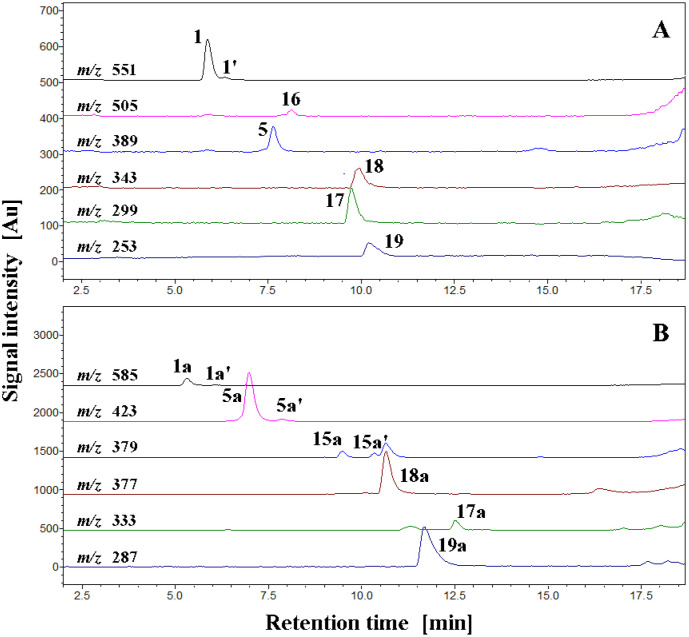
Chromatograms of NaOCl-induced (pH 7.4) betanin chlorination as well as oxidation, deglucosylation, and decarboxylation products registered in selected ion monitoring mode. The ions of non-chlorinated pigments (**A**) and chlorinated derivatives (**B**) are indicated.

**Figure 5:**
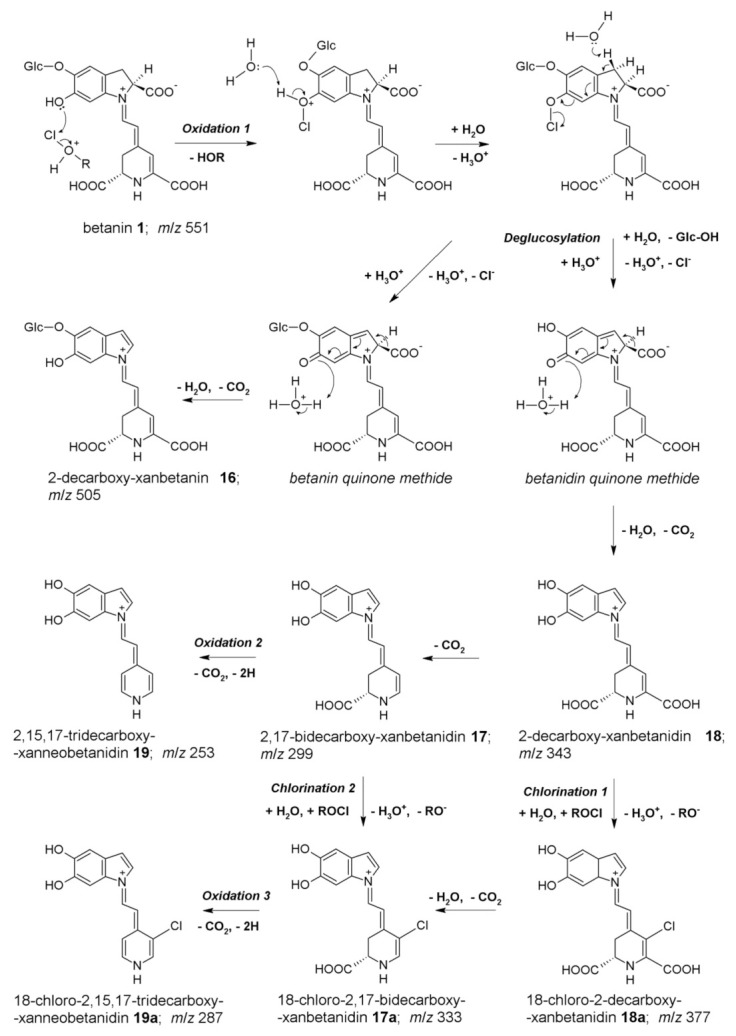
Proposed mechanism of betanin oxidation by NaOCl with subsequent alternate pathways of deglucosylation, decarboxylation, and chlorination steps based on this contribution and previous study [5].

Therefore, interpretation of the results of the anti-hypochlorite activity measurements should take into account that scavenging of hypochlorous acid by betalains proceeds according to different mechanisms and is especially different at pH 3 and at 7.4. Betalains exhibit a high tendency to be chlorinated at pH 3 but at pH 7.4, their rapid oxidation by hypochlorous acid is observed [5]. In addition, according to the presented results on chlorination and oxidation of betanin by NaOCl at pH 7.4 (Table 2, Figure 4), a multipath of the reactions can be deduced (Figure 5). 

The chromatograms of selected ions corresponding to the products of betanin chlorination and oxidation are depicted in Figure 4A,B and support the presence of oxidized derivatives not only of betanin but also of betanidin in the reaction mixtures at pH 7.4. Therefore, oxidation of betanin 1 results in the formation of 2-decarboxy-xanbetanin **16** at the first typical step observed for betacyanins [24] but at the same time, generation of the deglucosylated derivative 2-decarboxy-xanbetanidin **18** is detected. The deglucosylation of **1** presumably results from the formation of the quinonoid system during the oxidation of betanin which influences the stability of the 5-*O*-glucosidic bond. This phenomenon is analogous to the deglucosylation of gomphrenin during its oxidation by ABTS cation radicals [27]. As in the case of gomphrenin oxidation [27], this should be taken into account during the anti-hypochlorite activity measurements since the occurrence of betanidin or its derivatives as potent antioxidants in the reaction mixtures may be responsible for increasingly potentiating the high overall activity of betalains.

An inspection of the selected ion chromatograms (Table 2, Figure 4) and subsequent interpretation of the resulting chromatographic peak characteristics suggested a further scheme of reaction pathways (Figure 5) for the deglucosylated intermediate, 2-decarboxy-xanbetanidin **18**. Said scheme comprises decarboxylation steps alternately with oxidation and/or chlorination by HOCl (or ROCl in general) giving rise to the following chromophoric structures (confirmed by on-line DAD detection) 2,17-bidecarboxy-xanbetanidin **17** (*m/z* 299) and 2,15,17-tridecarboxy-xanneobetanidin **19** (*m/z* 253) as well as the chlorinated derivatives, 18-chloro-2-decarboxy-xanbetanidin **18a** (*m/z* 377), 18-chloro-2,17-bidecarboxy-xanbetanidin **17a** (*m/z* 333), and 18-chloro-2,15,17-tridecarboxy-xanneobetanidin **19a** (*m/z* 287). 

The detected ions fit to the oxidation pathway of betanidin **7** studied previously [24]; however, in this case, it is implemented in the whole representative reaction scheme for betanin **1** transformations (Figure 5).

### 2.3. Determination of Betalainic Reactivity against Hypochlorites by Measurements of Anti-Hypochlorite Activity with the Use of HCSe and HCS Fluorescent Probes 

Further study on the chlorination kinetics was possible to perform using in vitro anti-hypochlorite activity measurements of pure isolated betalainic pigments from the red beetroot extract (BRE). This gave a detailed insight to the differences of the chlorination ability of HOCl towards different pigments at selected pHs of the tested solutions but also into the combined effect with oxidation. 

Due to the fact that HOCl may be associated with various diseases, there is a growing need to develop a rapid and effective method for its detection and determination. Unfortunately, the development of novel methods for the detection of HOCl in vivo remains challenging due to its short half-life and the concurrent existence of various antioxidants within cells such as glutathione (GSH) and cysteine (Cys) [11,12]. In recent years, various analytical and bioanalytical methods, including traditional detection approaches such as chemiluminescence, colorimetric, electrochemical, and chromatographic techniques have been applied [11,12]. Among them, fluorescent methods exhibit more advantages in terms of selectivity, sensitivity, and spectral resolution. Fluorescent probes have shown promise as analytical tools for rapid and specific detection of HOCl/OCl^−^ [28,29]. Commonly utilized fluorescent probes for sensing HOCl are based on the skeleton of fluorescein [29,30,31,32], rhodamine [32,33,34], BODIPY (boron-dipyrromethene) [19,20,35,36,37], or coumarin [38,39]. Recently, HOCl fluorescent probes based on 1,8-naphthalimide [40] or phenothiazine [41] have also been designed and synthesized. 

**Figure 6:**
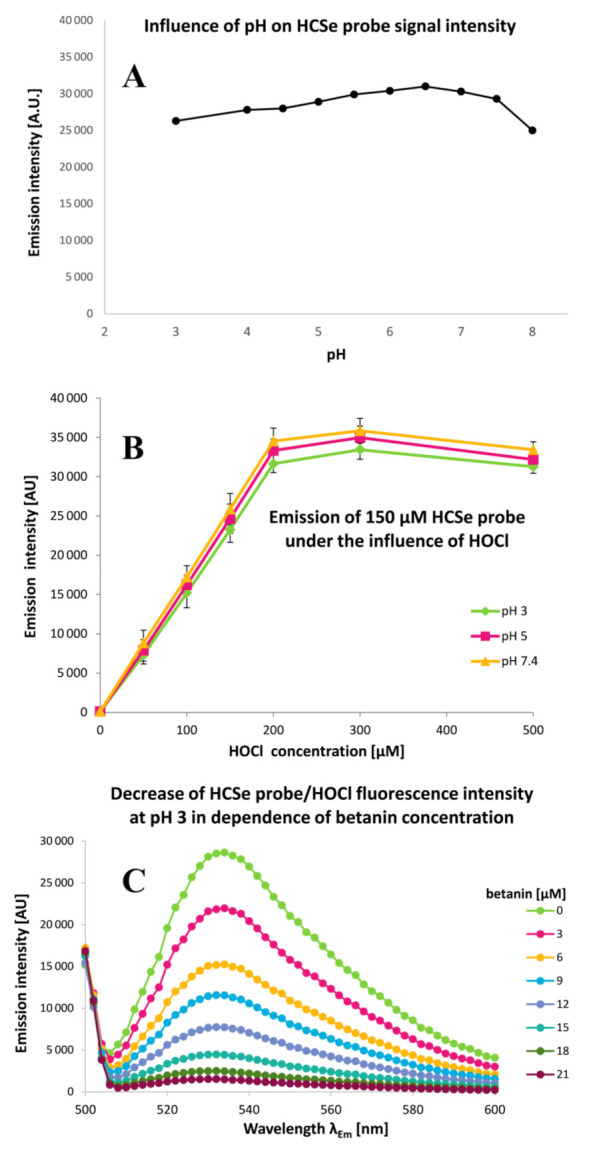
Dependence of the 150 μM HCSe fluorescent probe emission intensity (**A**) on the pH of buffers added to aqueous/acetonitrile solutions of 150 μM NaOCl at 25 °C. Linearity of the changes in fluorescence intensity of the HCSe probe (**B**) under the influence of increasing concentration of HOCl at pH 3, 5, and 7.4 (measured in triplicate) is presented at λ_Ex_ 480 nm and at λ_Em_ 536 nm. Decrease in the HCSe probe emission intensity (**C**) due to increasing concentration of betanin after 5 min of pigment incubation with 150 μM HOCl at pH 7.4 is shown in the emission spectra.

The HCSe and HCS probes’ high sensitivity to hypochlorous acid encouraged us to investigate their respective applicability in determination of betalains’ anti-hypochlorite activities. According to our results, these probes remained active, even when utilized within a more acidic environment (Figure 6). Consequently, in this study, our intention was to expand the working range of the probes to pH 3–7.4 in order to make it possible to test the anti-hypochlorite activity even when pH was as low as 3 when high chlorinating activity of NaOCl against betacyanins is observed [5,6]. Due to a much lower HOCl reaction rate observed for the HCS probe [19,20], the HCSe probe was selected for further studies wherein ca. 95 and 99.9% completion of the oxidation by HOCl was observed after 5 and 10 min, respectively. According to recent results [19,20], strong decreases in the emission of both the probes at higher pH (8–10) can be attributed to nearly complete ionization of HOCl, indicating that the reactive moiety for oxidation of the probes is HOCl but not OCl^−^ [9]. As mentioned above, this was at least partially confirmed in this study due to the fact that at more acidic pH, these probes are still active against HOCl (Figure 6). 

The studies on betalain anti-hypochlorite activity using the HCSe probe were performed for the BRE, its isolated betacyanins, betanin derivatives, and other selected antioxidants such as quercetin, catechin, caffeic acid, and ascorbic acid. The sensing performance of the HCSe probe was tested towards 150 μM NaOCl across a wide pH range. The fluorescence intensity of the reaction between HOCl and the HCSe is linear in the NaOCl concentration range of 0 to 200 μM (Figure 6). Therefore, the anti-hypochlorite activity tests related to 150 μM NaOCl were performed at pH 3, 5, and 7.4 in order to generate a comparison of the activities of the tested compounds in solutions containing meaningfully different composition of chlorine entities [5,6,9]. Based on previous results, partial conversion of HOCl to Cl_2_O in acidic conditions as well as the greater ability of Cl_2_O to chlorinate even at lower concentrations than HOCl should also be considered [9].

**Figure 7:**
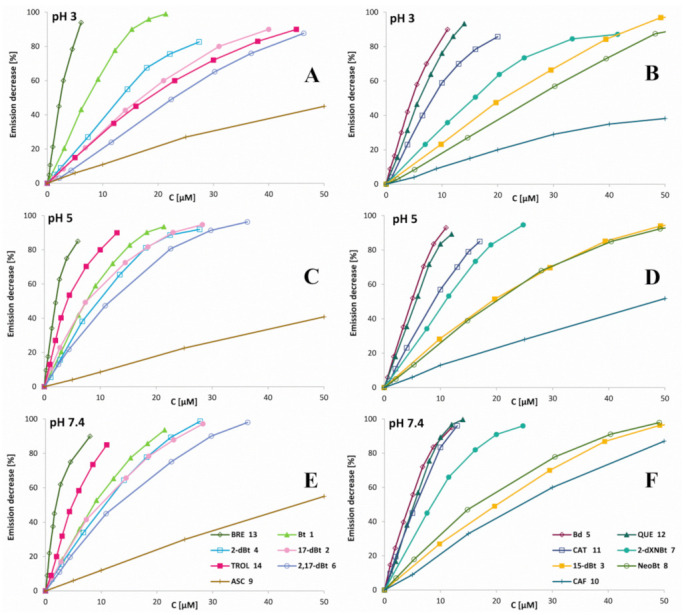
Percentile decrease in fluorescence intensity of the 150 μM HCSe probe (λ_Ex_ 480 nm, λ_Em_ 536 at 25 °C) added to 150 μM NaOCl solution after its 5 min incubation with BRE as well as analyzed pigments (betanin (**Bt**), 17-decarboxy-betanin (**17-dBt)**, 15-decarboxy-betanin (**15-dBt**), 2-decarboxy-betanin (**2-dBt**), betanidin (**Bd**), 2,17-decarboxy-betanin (**2,17-dBt**), neobetanin (**NeoBt**), and 2-decarboxy-xanneobetanin (**2-dXNBt**)), and selected antioxidants (ascorbic acid (**ASC**), caffeic acid (**CAF**), catechin (**CAT**) and quercetin (**QUE**)) as well as for the betalain-rich extract (**BRE**) and Trolox (**TROL**) of increasing concentration at pH 3 (**A**,**B**), 5 (**C**,**D**) and 7.4 (**E**,**F**). For clarity, only selected single measurement series were shown.

**Figure 8:**
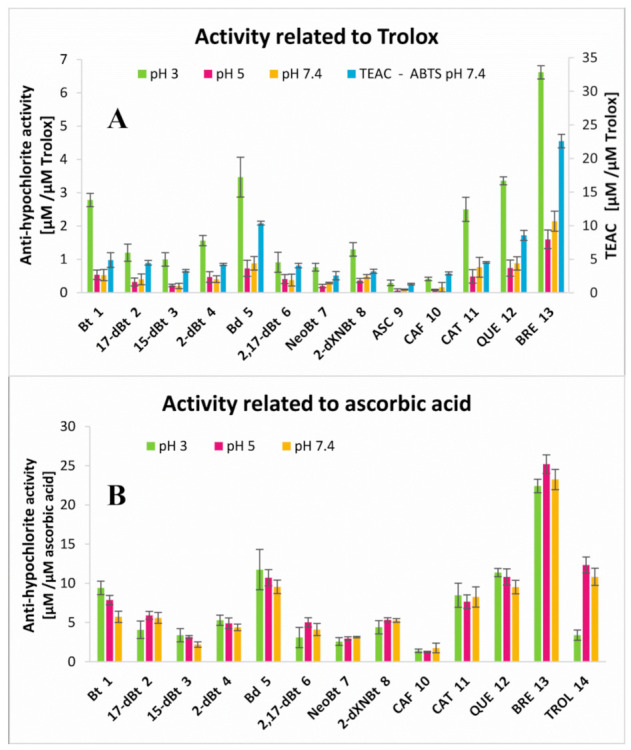
Anti-hypochlorite activity of tested betalain-rich extract (**BRE**) as well as betalainic pigments (betanin (**Bt**), 17-decarboxy-betanin (**17-dBt**), 15-decarboxy-betanin (**15-dBt**), 2-decarboxy-betanin (**2-dBt**), betanidin (**Bd**), 2,17-decarboxy-betanin (**2,17-dBt**), neobetanin (**NeoBt**), and 2-decarboxy-xanneobetanin (**2-dXNBt**)) and selected antioxidants (ascorbic acid (**ASC**), caffeic acid (**CAF**), catechin (**CAT**), and quercetin (**QUE**)) related to Trolox (**TROL**) (**A**) and ascorbic acid (**B**) evaluated using the HCSe probe at pH 3, 5, and 7.4 (25 °C) with comparison to the ABTS free radical scavenging activity (**A**) determined at pH 7.4 as Trolox equivalent antioxidant capacity (TEAC). The measurements were performed in triplicate.

As a result of the scavenging of hypochlorous acid by the selected pigments, a decrease in the intensity of fluorescence emission was observed for all of the reaction mixtures along with the increase in the analytes’ concentrations. The concentrations (C_50_) of the measured anti-hypochloric agents required for a 50% decrease in fluorescent emission of the 150 μM HCSe probe after 5 min of incubation with a reaction mixture of 150 μM NaOCl and the anti-hypochloric agent at 25 °C are presented in Table 3. Despite the betalains exhibiting significantly lower tendency of being chlorinated under physiological conditions compared to pH ranging from 3 to 5, a rapid elimination of hypochlorous acid at pH 7.4 was also noticed, presumably by betalains’ oxidation [5]. Fluorescence quenching for micro-molar concentrations of betalains as well as selected antioxidants is shown in Figure 7A–E. Based on the results of initial slope “a” factors’ measurements (Table 3) for fluorescence intensity-compound concentration dependence, the anti-hypochlorite activity (anti-HA) of the analytes related to Trolox (the water-soluble analogue of vitamin E), having a well-known antioxidant capacity and frequently used as a reference, was calculated (Figure 8, Table 3). The first inspection of the results (Figure 7 and Figure 8A, Table 3) enables us to conclude that the reactivity of Trolox with hypochlorous acid at pH 3 (Figure 7A,B) is much lower than at pH 5 (Figure 7C,D) and 7.4 (Figure 7E,F); therefore, relating the analytes’ activity to Trolox for comparison reasons in a wide pH range misleadingly increases the anti-HA values for all the tested compounds. Therefore, for this aim, a comparison of the analyte initial slope “a” factors to ascorbic acid (another antioxidant that exhibits the lowest activity) was also performed (Figure 8B) resulting in a decrease in anti-HA values at pH 3 to the levels observed at pH 5 and 7.4 for all the samples. The data suggest that all the tested compounds except Trolox can be applied as a reference in similar studies. In Figure 8A, the anti-HA values of the analytes were additionally compared to the antioxidant capacity for ABTS cation radicals determined at pH 7.4 as Trolox equivalent antioxidant capacity (TEAC). Comparison of these results allows for a conclusion that they all reasonably correlate even at pH 3; therefore, it more generally supports the oxidizing action of the hypochlorous acid rather than chlorination as a means of their scavenging by the analytes. 

Betanin **1** is capable of suppressing the fluorescence of the 150 μM HCSe probe at physiological pH 7.4 and phagosomal pH 5 in the presence of 150 μM NaOCl after 5 min of incubation at room temperature (Figure 7 and Figure 8A). It is worth noting that the required concentration of betanin pigment for complete removal of 150 μM OCl^−^ ranges from 0 to 20 μM at any tested pH (Figure 7). This indicates the high capability of betanin to scavenge hypochlorous acid well beyond the consequence of pigment chlorination even at pH 3 [5].

Betanidin **2** is even more effective in the removal of HOCl than **1** as a result of its reactive catechol moiety which is confirmed by the TEAC value. Betanidin **2** concentration, required for complete suppression of the fluorescence at pH 7.4, is almost two times lower than in the case of its glycosylated derivative **1** (Figure 7 and Figure 8A). The anti-hypochlorite activity of 17-decarboxy-betanin **2**, 2-decarboxy-betanin **4**, 2,17-bidecarboxy-betanin **6**/**6′**, and 2-decarboxy-xanneobetanin **8** are at similar levels and are slightly lower in relation to betanin **1** in contrast to 15-decarboxy-betanin **3**. Lower activity was observed for neobetanin **7**, which may result from the partial oxidation of the molecule (Figure 1) at carbons C-14,15 and thus, may lead to a completely different mechanism of chlorination/oxidation reaction as compared to the one previously stated in studies with NaOCl [5], where the neo-derivative **7** of betanin did not undergo chlorination. As a consequence, the anti-hypochlorite activity of **7** should be attributed to other chemical transformations.

For comparative purposes, four well-known antioxidants were also analyzed under the same reaction conditions. In comparison to betanidin **5**, much lower activity for ascorbic acid **9** and caffeic acid **10** was observed, while catechin **11** and quercetin **12** exhibit similar or slightly lower anti-hypochlorite and antioxidant properties (Figure 7 and Figure 8B, Table 3). 

The highest anti-hypochlorite activity and antioxidant capacity, and far exceeding the activity all of the betalain derivatives and selected antioxidants, were determined for the BRE. Its ability to scavenge hypochlorous acid is approximately twice the activity of the strongest pigment, betanidin **5**, at all of the tested pH values (Figure 7 and Figure 8, Table 3). A precise explanation of such high BRE activity requires further analytical research. Its higher activity could possibly be due to the composition of the extract containing some synergistic group of betalain derivatives that influences the overall activity of BRE. Additionally, other still unidentified or unknown compounds that occur naturally in red beetroot may be present in the extract. In any event, the superior anti-HOCl activity of BRE reported here must be ascribed to inherent structures present in BRE that possess rapid electron exchange potential with reducing agents such as HOCl or other ROS. In our previous studies, we have demonstrated that the metabolism of betalains in the human body results in structures with similar potential to the ones elucidated in this study. Logically, it follows that BRE and/or its fractions may serve as readily oxidizable decoys that could potentially be employed to protect biological tissues and cells from inflammatory insult from HOCl generated by chronic inflammation.

## 3. Materials and Methods 

### 3.1. Reagents

Sodium hypochlorite solution, diammonium salt of ABTS (2,2′-azino-bis(3-ethylbenzothiazoline-6-sulfonic acid)), almond β-glucosidase, ascorbic acid, caffeic acid, catechin, quercetin, Trolox, ethanol, acetonitrile, formic acid, acetone (HPLC grade), methanol (MS grade), deuterated trifluoroacetic acid, and D_2_O were obtained from Sigma Chemical Co. (St. Louis, MO, USA). HCSe and HCS probes were synthesized in the Department of Applied Chemistry, National Chiao Tung University, Hsinchu, Taiwan, ROC according to previously published methods [19,20]. BRE was obtained from FutureCeuticals, Inc. (Momence, IL, USA) [17].

### 3.2. Isolation and Preparation of Betalains from B. vulgaris Extracts 

For determination of the ability of certain betalains, and the BRE as a whole, to react with hypochlorous acid, the following pigments (Figure 1) were derived directly from the BRE by semipreparative chromatography: betanin **1**, 17-decarboxy-betanin **2**, 15-decarboxy-betanin **3**, neobetanin **7**, and 2-decarboxy-xanneobetanin **8 [17]**. Other pigments present at lower concentrations in the BRE (2-decarboxy-betanin **4** and 2,17-bidecarboxy-betanin/-isobetanin **6**/**6′**) were obtained by heating previously isolated betanin according to already-known procedures [21] or, in the case of betanidin, by enzymatic hydrolysis of betanin [24].

For the direct isolation of pigments from BRE, 10 g of the extract was dissolved in 10 L of water and was initially purified by ion-exchange chromatography. Obtained fractions were subjected to further semi-preparative HPLC purification. In order to obtain betanidin **5**, purified betanin was subjected to enzymatic deglycosylation catalyzed by almond *β*-glycosidase and further preparative fractionation in accordance with previously developed protocols [24]. 2-decarboxy-betanin **4** and 2,17-bidecarboxy-betanin/-isobetanin **6**/**6′** were obtained by thermal degradation of aqueous and ethanolic solutions of purified betanin acidified with glacial acetic acid at 85 °C (aqueous solution) and 75 °C (ethanolic solution) in a water bath for 40 min according to a previously published procedure [21].

### 3.3. Preparative Oxidation of Betanin and Neobetanin by ABTS Cation Radicals 

In order to obtain 2-decarboxy-xanneobetanin **8**, ABTS cation radicals generated from ABTS salt were gradually added to 500 mL of solution containing 50 mg of betanin **1** in 10 mM acetate buffer of pH 5. The solution was stirred on a magnetic stirrer and the absorption at λ_max_ 538 nm was measured until it was diminished three times. After the next 10 min, the mixture was subjected to isolation and purification using the ion-exchange chromatographic system and semipreparative HPLC.

### 3.4. Semi-Preparative HPLC Purification of Betalain Fractions 

Preliminary purification of the pigments was performed by flash chromatography on a column 30 × 40 mm filled with Sepra™ ZT-SAX 30 μm polymer, 85 Å (Phenomenex, Torrance, CA, USA). Further separation and isolation of pigments were performed on a HPLC semipreparative column Luna C18(2) 250 × 20 mm i.d., 10 μm (Phenomenex) with a 20 × 25 mm i.d. guard column of the same material (Phenomenex). A gradient system consisting of 1% aqueous formic acid (solvent A) and acetone (solvent B) for semi-synthesized betanidin **5** was used as follows: 0 min, 10% B; increasing to 10 min, 12% B; increasing to 20 min, 14% B; increasing to 30 min, 70% B and for betanin was used as follows: 0 min, 10% B; increasing to 10 min, 11% B; increasing to 20 min, 12% B; increasing to 30 min, 13% B; increasing to 40 min; 70% B. The injection volume was 15 mL with a flow rate of 20 mL/min. Detection was performed using a PDA UV/Vis detector at 538, 505, 480, and 440 nm, at a column temperature of 22 °C. The eluates were pooled and concentrated under reduced pressure at 25 °C and finally, freeze-dried. All the solutions were concentrated in rotary evaporators at 25 °C under reduced pressure to remove the organic solvent and stored at −20 °C for further assays.

### 3.5. Chromatographic Analysis by LC-DAD-ESI-MS/MS System

For the chromatographic and mass spectrometric analyses, an LCMS-8030 mass spectrometric system (Shimadzu, Kyoto, Japan) coupled to LC-20ADXR HPLC pumps, an SIL-20ACXR injector model, and a PDA (photo diode array) detector model SPD-M20A, all controlled with LabSolutions software version 5.60 SP1 (Shimadzu) was used. The samples were eluted through a 150 × 4.6 mm i.d., 5.0 μm, Kinetex C18 chromatographic column preceded by a guard column of the same material (Phenomenex, Torrance, CA, USA). The injection volume was 20 μL and the flow rate was 0.5 mL/min. The column was thermostated at 40 °C. The separation of the analytes was performed with a binary gradient elution. For the separation, a gradient system consisting of 2% aqueous formic acid (solvent A) and pure methanol (solvent B) was used as follows: 0 min, 5% B; increasing linearly to 12 min, 30% B; increasing linearly to 15 min, 80% B. The full range of PDA signals was recorded and chromatograms were individually displayed at 538, 505, 490, and 440 nm. Positive ion electrospray mass spectra were recorded on the LC-MS system, which was controlled with LabSolutions software for registration of total ion chromatograms, mass spectra, and ion chromatograms in selected ion monitoring mode (SIM) as well as the fragmentation spectra. The ionization electrospray source was operated in positive mode (ESI+), at an electrospray voltage of 4.5 kV and capillary temperature at 250 °C, using N_2_ as a gas for the spray. Argon was used as the collision gas for the collision-induced dissociation (CID) experiments. The relative collision energies for MS/MS analyses were set at −35 V.

### 3.6. HCSe and HCS Assays for Determination of Anti-Hypochlorite Activity 

For determination of the ability of betalains and BRE to scavenge hypochlorous acid, pigment solutions of gradually increasing concentrations were dosed to wells of the black 96-well plate (Nunc™ F96 MicroWell™ Black Polystyrene Plate, Thermo Scientific™, Waltham, MA, USA) buffered with 25 mM acetate (pH 3 and 5) and phosphate (pH 7.4) buffers. Afterwards, an acetonitrile/aqueous solution of NaOCl was added to the wells and the final concentration was 150 μM in 200 μL of the total sample volume. After 5 min of chlorination reaction, 60 μL of 500 μM HCSe or HCS acetonitrile solution was dosed into the wells for their final concentration of 150 μM. Then, the plate was shaken for 20 s and the fluorescence was measured in a Tecan Infinite 200 microplate reader (Tecan Austria GmbH, Grödig/Salzburg, Austria). The HCSe and HCS probes were excited at λ_Exc_ 480 nm and the emission signal was monitored at λ_Em_ 536 and 515 nm, respectively. Then, fluorescence spectra were recorded for all wells at the range of λ 500–650 nm, excited by light of λ_Exc_ 480 nm. Similar measurements were performed for selected, commonly known antioxidants including: ascorbic acid **9** (ASC), caffeic acid **10** (CAF), catechin **11** (CAT), and quercetin **12** (QUE). The concentrations of measured pigments as well as selected antioxidants were individually set for each sample so that the fluorescence decrease in the probes was in the range of 10–90% of their initial value in the sample without tested analytes. The results for all tested compounds were referenced to a standard of Trolox as well as ascorbic acid. All measurements were repeated in triplicate. 

### 3.7. Measurement of Antioxidant Activity with ABTS Cation Radicals 

The antioxidant activity of the tested BRE and isolated compounds was measured by the Trolox equivalent antioxidant capacity (TEAC) assay based on their reaction with ABTS cation radicals [23] in 96-well plates of a Tecan Infinite 200 microplate reader (Tecan Austria GmbH, Grödig/Salzburg, Austria). ABTS cation radicals were generated from ABTS salt by reaction of 2.45 mM potassium persulfate with 7 mM ABTS salt in 0.002 M phosphate-buffered saline at pH 7.4 for 16 h at room temperature in the dark. The resultant ABTS cation radical solution was diluted with water and adjusted to absorption of 0.7 at 734 nm for the experiments. Just before the measuring step, 10 µL of the measured sample dilutions were individually loaded into wells containing 190 µL of ABTS cation radicals. Then, the mixtures were shaken for 20 s by a shaker within the reader. The absorption measurement at 734 nm was performed after 30 min of reaction at 25 °C. All experiments were performed in triplicate.

## 4. Conclusions

The results of this study show that all of the tested betalainic pigments are efficient in the removal of hypochlorous acid; however, betanidin exhibited the highest activity. Additionally, the fact that betalains as well as the BRE inactivated HOCl within a few minutes, even at micro-molar concentrations, emphasizes their highly efficient anti-hypochlorite activity and possibility for applications in physiological conditions wherein HOCl has a very short half-life. In combination with the ability of fluorescent probes to penetrate cell membranes, BRE may also be used as a scavenging factor to remove HOCl in future in vitro trials. Finally, and of special interest, is the fact that BRE significantly and unexpectedly outperformed any of the individual pigments in terms of HOCl scavenging ability. As such, these results further support the use of BRE as a natural dietary supplement that may provide support against chronic inflammation. 

## Figures and Tables

**Table 1:** Spectrophotometric and low-resolution mass spectrometric data of the studied betanidin, betanin, and its derivatives for their anti-hypochlorite activity. For the chromatographic profile of the pigments in the BRE, refer to a previous study [17].

No.	Compound	λ_max_ [nm]	*m/z* [M+H]^+^	*m/z* from MS/MS of [M+H]^+^
**1**	betanin	538	551	389
**2**	17-decarboxy-betanin	505	507	345
**3**	15-decarboxy-betanin	528	507	345
**4**	2-decarboxy-betanin	533	507	345
**5**	betanidin	540	389	345
**6/6′**	2,17-bidecarboxy-betanin/-isobetanin	507	463	301
**7**	neobetanin	466	549	387; 343; 299
**8**	2-decarboxy-xanneobetanin	422	503	341; 297; 253

**Table 2:** Chromatographic, spectrophotometric, and mass spectrometric data of the products of betanin oxidation, chlorination, deglucosylation, and decarboxylation induced by NaOCl at pH 7.4.

No.	Compound	R_t_ [min]	λ_max_ [nm]	*m/z* [M+H]^+^	*m/z* from MS/MS of [M+H]^+^
	Betanin oxidation, deglucosylation, and decarboxylation products formed at pH 7.4				
**1**	betanin	5.9	538	551	389
**1′**	isobetanin	6.3	538	551	389
**5**	betanidin	7.7	540	389	345
**15**	17-decarboxy-betanidin	7.8	511	345	299; 255
**15′**	17-decarboxy-isobetanidin	8.4	511	345	299; 255
**16**	2-decarboxy-xanbetanin	8.2	445	505	343; 297
**17**	2,17-bidecarboxy-xanbetanidin	9.7	467	299	255
**18**	2-decarboxy-xanbetanidin	9.9	490	343	299; 255
**19**	2,15,17-tridecarboxy-xanneobetanidin	10.2	401	253	253
	Betanin chlorination products formed at pH 7.4				
**1a**	18-chloro-betanin	5.3	522	585	423; 387
**1a’**	18-chloro-isobetanin	6.1	522	585	423; 387
**5a**	18-chloro-betanidin	7.0	527	423	387; 341
**5a’**	18-chloro-isobetanidin	7.8	527	423	387; 341
**15a**	18-chloro-17-decarboxy-betanidin	9.5	525	379	343; 333
**15a’**	18-chloro-17-decarboxy-isobetanidin	10.3	525	379	343; 333
**17a**	18-chloro-2,17-bidecarboxy-xanbetanidin	12.5	499	333	287; 253
**18a**	18-chloro-2-decarboxy-xanbetanidin	10.7	494	377	331; 287; 253
**19a**	18-chloro-2,15,17-tridecarboxy-xanneobetanidin	11.7	401	287	251

**Table 3:** Anti-hypochloric activity (Anti-HA) of betanidin (**Bd**), betanin (**Bt**), and its derivatives (17-decarboxy-betanin (**17-dBt)**, 15-decarboxy-betanin (**15-dBt**), 2-decarboxy-betanin (**2-dBt**), betanidin (**Bd**), 2,17-decarboxy-betanin (**2,17-dBt**), neobetanin (**NeoBt**), and 2-decarboxy-xanneobetanin (**2-dXNBt**)) and selected antioxidants (ascorbic acid (**ASC**), caffeic acid (**CAF**), catechin (**CAT**) and quercetin (**QUE**)) related to Trolox (**TROL**) based on measurements of initial slope “a” factors for fluorescence intensity-compound concentration dependence at selected pH. C50—concentration of the measured anti-hypochloric agent required for a 50% decrease in the fluorescence emission of the 150 mM HCSe probe after 5 min of its incubation with reaction mixture of 150 mM NaOCl and the anti-hypochloric agent at 25 °C. The measurements were performed in triplicate.

Compound	C_50_ [μM]						“a” Factor of Initial Slope						Anti-HA					
(No.)	pH 3		pH 5		pH 7.4		pH 3		pH 5		pH 7.4		pH 3		pH 5		pH 7.4	
Bt **(1)**	7.37	±0.4	7.20	±0.4	8.14	±0.9	6.72	±0.4	6.49	±0.4	5.75	±0.4	2.78	±0.1	0.54	±0.3	0.50	±0.1
17-dBt **(2)**	15.5	±1.1	5.82	±0.6	8.66	±1.1	2.91	±1.1	8.15	±0.5	5.61	±0.1	1.20	±0.1	0.32	±0.3	0.40	±0.3
15-dBt **(3)**	20.6	±1.4	19.0	±1.9	22.6	±2.4	2.41	±0.2	2.62	±0.2	2.20	±0.2	1.00	±0.1	0.22	±0.1	0.19	±0.0
2-dBt **(4)**	10.9	±0.3	8.11	±0.9	11.2	±1.3	3.78	±0.3	5.67	±0.4	4.40	±0.3	1.56	±0.1	0.47	±0.2	0.38	±0.2
Bd **(5)**	5.37	±0.3	5.26	±0.7	4.95	±0.5	8.38	±0.7	8.85	±1.1	9.59	±1.0	3.47	±0.3	0.73	±0.5	0.83	±0.1
2,17-dBt **(6)**	19.9	±1.4	9.77	±1.0	12.1	±1.4	2.21	±0.5	4.95	±0.2	4.12	±0.4	0.91	±0.2	0.41	±0.2	0.36	±0.2
NeoBt **(7)**	25.8	±3.0	20.1	±2.0	15.8	±1.6	1.85	±0.1	2.45	±0.1	3.16	±0.2	0.76	±0.1	0.20	±0.0	0.27	±0.2
2-dXNBt **(8)**	13.9	±1.1	11.0	±1.1	9.26	±0.9	3.14	±0.2	4.42	±0.4	5.30	±0.5	1.30	±0.1	0.37	±0.3	0.46	±0.3
ASC **(9)**	54.6	±5.3	50.6	±4.3	42.7	±3.3	0.71	±0.6	0.83	±0.1	1.00	±0.0	0.30	±0.2	0.07	±0.0	0.09	±0.0
CAF **(10)**	18.8	±2.0	28.5	±2.0	28.2	±2.0	1.00	±0.8	1.02	±1.0	1.76	±0.1	0.41	±0.3	0.08	±0.0	0.15	±0.1
CAT **(11)**	7.10	±0.6	7.18	±0.6	6.00	±0.4	6.04	±0.5	5.92	±0.5	8.29	±0.6	2.50	±0.2	0.49	±0.2	0.72	±0.2
QUE **(12)**	5.75	±0.8	5.35	±0.8	5.21	±0.5	8.11	±0.7	8.93	±0.8	9.56	±0.8	3.36	±0.2	0.74	±0.5	0.83	±0.6
BRE **(13)**	2.94	±0.3	2.19	±0.3	1.99	±0.1	16.0	±1.2	19.4	±1.9	23.3	±1.6	6.62	±0.3	1.60	±0.1	2.02	±0.2
TROL **(14)**	18.6	±1.3	3.72	±1.3	3.91	±0.3	2.42	±0.3	12.1	±1.0	10.9	±0.9	1.00	±0.0	1.00	±0.0	1.00	±0.0

## Data Availability

Not applicable.

## References

[1] Khan M.I., Giridhar P. (2015). Plant betalains: Chemistry and biochemistry. Phytochemistry.

[2] Azeredo H.M.C. (2009). Betalains: Properties, sources, applications, and stability—A review. Int. J. Food Sci. Tech..

[3] Gandía-Herrero F., Escribano J., García-Carmona F. (2016). Biological activities of plant pigments betalains. Crit. Rev. Food Sci..

[4] Khan M.I. (2015). Plant betalains: Safety, antioxidant activity, clinical efficacy, and bioavailability. Compr. Rev. Food Sci. Food Saf..

[5] Wybraniec S., Starzak K., Pietrzkowski Z. (2016). Chlorination of betacyanins in several hypochlorous acid systems. J. Agric. Food Chem..

[6] Wybraniec S., Starzak K., Szneler E., Pietrzkowski Z. (2016). Separation of chlorinated diastereomers of decarboxy-betacyanins in myeloperoxidase catalyzed chlorinated *Beta vulgaris* L. extract. J. Chromatogr. B.

[7] Pietrzkowski Z., Nemzer B., Michałowski T., Wybraniec S. (2010). Influence of betalain-rich extract on reduction of discomfort associated with osteoarthritis. New Med..

[8] Pietrzkowski Z., Argumedo R., Shu C., Nemzer B., Wybraniec S., Reyes-Izquierdo T. (2014). Betalain-rich red beet concentrate (BRC) improves knee discomfort and function: A double blind, placebo-controlled clinical study. Nutr. Diet. Suppl..

[9] Kettle A.J., Albrett A.M., Chapman A.L., Dickerhof N., Forbes L.V., Khalilova I., Turner R. (2014). Measuring chlorine bleach in biology and medicine. Biochim. Biophys. Acta Gen. Subj..

[10] Klebanoff S.J. (2005). Myeloperoxidase: Friend and foe. J. Leukoc. Biol..

[11] Zhang R., Song B., Yuan J. (2018). Bioanalytical Methods for Hypochlorous Acid Detection: Recent Advances and Challenges. Trends Anal. Chem..

[12] Albrich J.M., McCarthy C.A., Hurst J.K. (1981). Biological reactivity of hypochlorous acid: Implications for microbicidal mechanisms of leukocyte myeloperoxidase. Proc. Natl. Acad. Sci. USA.

[13] Dennis W.H., Olivieri V.O., Krusé C.W. (1979). The reaction of nucleotides with aqueous hypochlorous acid. Water Res..

[14] Visser M.C.M., Winterbourn C.C. (1991). Oxidative damage to fibronectin: I. The effects of the neutrophil myeloperoxidase system and HOCl. Arch. Biochem. Biophys..

[15] Carr A.C., Vissers M.C.M., Domigan N.M., Winterbourn C.C. (1997). Modification of red cell membrane lipids by hypochlorous acid and haemolysis by preformed lipid chlorohydrins. Redox Rep..

[16] Allegra M., Furtmuller P.G., Jantschko W., Zederbauer M., Tesoriere L., Livrea M.A., Obinger C. (2005). Mechanism of interaction of betanin and indicaxanthin with human myeloperoxidase and hypochlorous acid. Biochem. Biophys. Res. Commun..

[17] Nemzer B., Pietrzkowski Z., Spórna A., Stalica P., Thresher W., Michałowski T., Wybraniec S. (2011). Betalainic and nutritional profiles of pigment-enriched red beet root (*Beta vulgaris* L.) dried extracts. Food Chem..

[18] Kumorkiewicz A., Starzak K., Sutor K., Nemzer B., Pietrzkowski Z., Popenda Ł., Wybraniec S. (2020). Structural Study on Hypochlorous Acid-Mediated Chlorination of Betanin and Its Decarboxylated Derivatives from an Anti-Inflammatory *Beta vulgaris* L. Extract. Molecules.

[19] Liu S.R., Wu S.P. (2013). Hypochlorous acid turn-on fluorescent probe based on oxidation of diphenyl diselenide. Org. Lett..

[20] Liu S.R., Vedamalai M., Wu S.P. (2013). Hypochlorous acid turn-on boron dipyrromethene probe based on oxidation of methyl phenyl sulphide. Anal. Chim. Acta.

[21] Wybraniec S. (2005). Formation of decarboxylated betacyanins in heated purified betacyanin fractions from red beet root (*Beta vulgaris* L.) monitored by LC-MS/MS. J. Agric. Food Chem..

[22] Nilsson T. (1970). Studies into the pigments in beetroot (*Beta vulgaris* L. ssp. *vulgaris var. rubra* L.). Lantrukshogsk. Ann..

[23] Walker R.B., Everette J.D. (2009). Comparative reaction rates of various antioxidants with ABTS radical cation. J. Agric. Food Chem..

[24] Wybraniec S., Michałowski T. (2011). New pathways of betanidin and betanin enzymatic oxidation studied by LC-DAD-ESI-MS/MS. J. Agric. Food Chem..

[25] Wybraniec S., Starzak K., Skopinska A., Nemzer B., Pietrzkowski Z., Michalowski T. (2013). Studies on nonenzymatic oxidation mechanisms in neobetanin, betanin, and decarboxylated betanins. J. Agric. Food Chem..

[26] Kumorkiewicz A., Szmyr N., Popenda Ł., Pietrzkowski Z., Wybraniec S. (2019). Alternative mechanisms of betacyanin oxidation by complexation and radical generation. J. Agric. Food Chem..

[27] Kumorkiewicz A., Szneler E., Wybraniec S. (2018). Conjugation of Oxidized Betanidin and Gomphrenin Pigments from *Basella alba* L. Fruits with Glutathione. J. Agric. Food Chem..

[28] Zhang Y.R., Liu Y., Feng X., Zhao B.X. (2017). Recent progress in the development of fluorescent probes for the detection of hypochlorous acid. Sens. Actuat. B Chem..

[29] Jin X., Hao L., Hu Y., She M., Shi Y., Obst M., Li J., Shi Z. (2013). Two novel fluorescein-based fluorescent probes for hypochlorite and its real applications in tap water and biological imaging. Sens. Actuat. B Chem..

[30] Huo F.J., Zhang J.J., Yang Y.T., Chao J.B., Yin C.X., Zhang X.B., Chen T.G. (2012). A fluorescein-based highly specific colorimetric and fluorescent probe for hypochlorites in aqueous solution and its application in tap water. Sens. Actuat. B Chem..

[31] Setsukinai K.I., Urano Y., Kakinuma K., Majima H.J., Nagano T. (2003). Development of novel fluorescence probes that can reliably detect reactive oxygen species and distinguish specific species. J. Biol. Chem..

[32] Zhang Z., Zheng Y., Hang W., Yan X., Zhao Y. (2011). Sensitive and selective off-on rhodamine hydrazide fluorescent chemosensor for hypochlorous acid detection and bioimaging. Talanta.

[33] Koide Y., Urano Y., Hanaoka K., Terai T., Nagano T. (2011). Development of an Si-rhodamine-based far-red to near-infrared fluorescence probe selective for hypochlorous acid and its applications for biological imaging. Am. Chem. Soc..

[34] Kenmoku S., Urano Y., Kojima H., Nagano T. (2007). Development of a highly specific rhodamine-based fluorescence probe for hypochlorous acid and its application to real-time imaging of phagocytosis. J. Am. Chem. Soc..

[35] Park J., Kim H., Choi Y., Kim Y. (2013). A ratiometric fluorescent probe based on a BODIPY–DCDHF conjugate for the detection of hypochlorous acid in living cells. Analyst.

[36] Kang J., Huo F., Yue Y., Wen Y., Chao J., Zhang Y., Yin C. (2017). A solvent depend on ratiometric fluorescent probe for hypochlorous acid and its application in living cells. Dye. Pigment..

[37] Xu X.X., Qian Y. (2017). A novel pyridyl triphenylamine–BODIPY aldoxime: Naked-eye visible and fluorometric chemodosimeter for hypochlorite. Spectrochim. Acta Mol. Biomol. Spectrosc..

[38] Wang L., Li W., Zhi W., Ye D., Zhang W., Ni L. (2018). Rapid detection of hypochlorite by a coumarin-based hydrazide in aqueous solution and its application in live-cell imaging. Sens. Actuat. B Chem..

[39] Song X., Dong B., Kong X., Wang C., Zhang N., Lin W. (2018). Construction of a ratiometric fluorescent probe with an extremely large emission shift for imaging hypochlorite in living cells. Spectrochim. Acta Mol. Biomol. Spectrosc..

[40] Zhang P., Wang H., Zhang D., Zeng X., Zeng R., Xiao L., Tao H., Long Y., Yi P., Chen J. (2018). Two-photon fluorescent probe for lysosome-targetable hypochlorous acid detection within living cells. Sens. Actuat. B Chem..

[41] Deng B., Ren M., Kong X., Zhou K., Lin W. (2018). Development of an enhanced turn-on fluorescent HOCl probe with a large Stokes shift and its use for imaging HOCl in cells and zebrafish. Sens. Actuat. B Chem..

